# Brain injury, endocrine disruption, and immune dysregulation in HIV-positive men who have sex with men with late HIV diagnosis

**DOI:** 10.3389/fimmu.2025.1436589

**Published:** 2025-03-19

**Authors:** Yihui He, Hao Liu, Meixin Ren, Gaungqiang Sun, Yundong Ma, Miaotian Cai, Rui Wang, Lei Wang, Tong Zhang, Yang Zhang

**Affiliations:** ^1^ Postgraduate Union Training Base of Jinzhou Medical University, PLA Rocket Force Characteristic Medical Center, Beijing, China; ^2^ Department of Neurology, PLA Rocket Force Characteristic Medical Center, Beijing, China; ^3^ Center for Infectious Disease, Beijing Youan Hospital, Capital Medical University, Beijing, China; ^4^ Beijing Institute for Sexually Transmitted Disease Control, Beijing, China; ^5^ Beijing Key Laboratory of Mental Disorders, National Clinical Research Center for Mental Disorders and National Center for Mental Disorders, Beijing Anding Hospital, Capital Medical University, Beijing, China; ^6^ Advanced Innovation Center for Human Brain Protection, Capital Medical University, Beijing, China; ^7^ Department of Respiratory and Critical Care Medicine, Beijing Youan Hospital, Capital Medical University, Beijing, China

**Keywords:** human immunodeficiency virus (HIV), late HIV diagnosis, multimodal magnetic resonance imaging, peripheral immunity, inflammation

## Abstract

**Background:**

In the realm of public health, late human immunodeficiency virus (HIV) diagnosis remains prevalent and is associated with neuropsychiatric adverse events. However, there is limited documentation regarding the impact of late HIV diagnosis (LD) on brain integrity, neurotrophic factors, endocrine function, and immunity in HIV-positive men who have sex with men (MSM).

**Methods:**

Participants (38 LD and 34 non-LD of MSM) underwent comprehensive infectious disease and psychiatric assessments, multimodal magnetic resonance imaging (MRI) scans, neurotrophic factors, endocrine, and immunological evaluations. Immune cell levels, along with peripheral plasma concentrations of neurotrophic factors and hormones, were measured using enzyme-linked immunosorbent assays and flow cytometry, respectively. T1-weighted images along with resting-state functional MRI were applied to assess brain function and structure while also examining correlations between imaging alterations and clinical as well as peripheral blood variables. The data for this study originated from a subset of the cohort in HIV-associated neuropsychiatric disorders research.

**Results:**

Compared to participants in the non-LD group, those in the LD group showed a lower total gray matter volume (GMV), with reduced GMV primarily observed in the left supramarginal gyrus. Participants in the LD group exhibited differences in brain function with certain regions and decreased functional connectivity between these altered regions and connected structures. A two-way factorial analysis of variance examining the main effects and interactions between groups and neuropsychiatric disorders revealed significant main effects of LD on specific brain regions. Furthermore, we found that individuals in the LD group had higher levels of cortisol, a lower frequency of central memory T cells, and elevated expression levels of perforin in double-negative T cells. These imaging findings were significantly correlated with endocrine, immune, and clinical variables.

**Conclusion:**

This study suggests that LD may contribute to brain injury, endocrine disruption, and immune dysregulation in HIV-positive MSM. Consequently, there is an urgent need to develop public health strategies targeting late diagnosis, with a focus on strengthening screening and early detection for high-risk populations, as well as monitoring brain injury, endocrine, and immune functions in individuals with LD, and formulating precise, individualized intervention strategies to reduce the long-term impact of LD on the health of HIV-positive MSM.

## 1 Introduction

Despite the widespread availability of human immunodeficiency virus (HIV) testing and antiretroviral therapy (ART), diagnosing HIV at an advanced stage of the disease remains a key challenge in the HIV epidemic (1). Factors contributing to late HIV diagnosis (LD) among people living with HIV (PLWH) may include infrequent routine screening practices, low perception of risk behaviors, and the lack of general social support for PLWH (2). LD has been widely linked to poor individual and community-level clinical outcomes. Delayed detection leads to postponed treatment initiation resulting in increased morbidity and mortality from acquired immunodeficiency syndrome (AIDS), treatment complexity, healthcare costs, and increased transmission because of unawareness of infection status (3, 4). Previous reports have demonstrated that lower CD4 nadirs are associated with a higher risk of neuropsychological impairment (5). However, limited data exist regarding the effect of LD on brain structure and function among PLWH.

Multimodal magnetic resonance imaging (MRI) has emerged as a crucial technique for the early detection of neuropathological changes in the brain (6). It has yielded remarkable insights into the neuropathology of HIV and may serve as an early indicator and marker of the effects of LD on brain structure and function (7). Previous research showed that PLWH exhibit reduced gray matter volume (GMV) and functional abnormalities compared to healthy controls (8). LD individuals often present with low CD4^+^ T cell counts (9), which have been correlated with smaller thalamic and hippocampal volumes, as well as larger ventricular volumes according to relevant studies (10, 11). Therefore, multimodal MRI has proven successful in examining changes in brain structure and function, offering superior accuracy and reliability compared to alternative methods (12, 13).

HIV infection is associated with chronic immune activation, systemic inflammatory responses, and dysregulation of the hypothalamic-pituitary-adrenal (HPA) axis function (14). These pathophysiological changes may be more pronounced in PLWH with LD, particularly among those with restricted immune recovery. Research indicates that individuals with LD, even when receiving ART, generally show poor immune recovery and are considered a high-risk group for immune non-responders (15, 16). Increasing evidence suggests a connection between changes in peripheral immune responses and alterations in brain structure and function (17–19). HIV infection induces chronic immune activation and systemic inflammation, which not only affect the peripheral immune system but also influence the central nervous system (CNS) via various pathways. For instance, HIV and its proteins (such as gp120 and Tat) are capable of crossing the blood-brain barrier, infecting microglia and astrocytes, and initiating neuroinflammation (20, 21). Additionally, the high levels of inflammatory factors (such as TNF-α, IL-1β) and free radicals produced by chronic inflammation can disrupt the blood-brain barrier, induce oxidative stress, and cause neuronal apoptosis, resulting in alterations in brain structure and function (22, 23).

In individuals with LD, the risk of brain injury is significantly increased due to lower CD4^+^ T cell counts and higher viral loads at diagnosis, leading to more severe immune dysfunction and stronger inflammatory responses. HIV infection and ART may interfere with HPA axis function, resulting in abnormal levels of hormones such as cortisol. Cortisol plays a critical role in immune regulation, and its imbalance can intensify immune dysregulation. Prolonged viremia and chronic inflammation in these patients may exacerbate this imbalance, rendering the immune system more fragile. Dysfunction of the HPA axis directly impacts brain function. Hormones such as cortisol and related neurotrophic factors are essential for neuronal survival, synaptic plasticity, and cognitive function (24). Long-term hormone imbalances may lead to atrophy in brain regions such as the hippocampus and prefrontal cortex, impairing memory and executive function (25, 26). For LD patients, late diagnosis of the disease may impair hormone regulation and neurorepair abilities, leading to more pronounced brain injury. Fragments and damage-associated molecular patterns produced by brain injury can serve as endogenous danger signals, activating microglia and the peripheral immune system, which further intensifies systemic inflammation and immune dysregulation. For LD individuals with immune non-reconstitution, breaking this feedback loop is more difficult, resulting in more severe systemic consequences. Neuroimaging evidence indicates that in the PLWH with comorbid anxiety disorders, regional homogeneity (ReHo) in the right superior parietal cortex is negatively correlated with the initial CD4^+^ T cell count and brain-derived neurotrophic factor (BDNF) levels (27). PLWH exhibit characteristic brain injury and disturbances in peripheral immunity, endocrinology, and neurotrophic factors, with related studies focusing on areas such as infection versus non-infection and treatment versus no treatment. However, research data on LD versus non-LD remains very limited.

Men who have sex with men (MSM) bear a disproportionate burden of HIV infection worldwide (28–30), but LD continues to be a persistent challenge for this population. Structural factors (such as stigma and legal barriers) often restrict their access to testing services (31, 32), while behavioral factors (including risk perception biases) may further exacerbate diagnostic delays (33).

This cross-sectional study aimed to investigate differences in brain structure and function between MSM with and without LD using multimodal MRI. We also examined the impact of LD on neurotrophic factors, endocrine hormones, and immune cells to better understand its effects on immunity, endocrine, and neurotrophins. Finally, correlation analyses were performed to evaluate this relationship between imaging alterations and clinical data as well as the above-mentioned peripheral blood indicators. This study may provide a potential theoretical basis and data support for future research on LD-related imaging abnormalities as well as their impact on endocrinology and immunity.

## 2 Materials and methods

### 2.1 Participants

The cross-sectional study was approved by the Institutional Ethics Committee of Beijing Youan Hospital, Capital Medical University. Prior to signing a written informed consent form, all participants were supplied with comprehensive information about the entire procedure and potential risks. This study used *International Classification of Diseases 11th revision (ICD-11)* and *Diagnostic and Statistical Manual of Mental Disorders, 5th edition* (DSM-5) for the classification and diagnosis of various diseases, symptoms, and health issues (34, 35). The inclusion criteria for our investigation were as follows: (1) Chinese HIV-positive MSM; (2) at least 18 years of age; and (3) right-handed individuals. The exclusion criteria included: (1) individuals with current or past opportunistic CNS infections; (2) individuals with a history of confounding neurological diseases including Parkinson’s disease, multiple sclerosis, epilepsy, or dementia; (3) previous head injury with loss of consciousness lasting more than half an hour; (4) the presence of space-occupying brain lesions; (5) individuals with MRI contraindications or claustrophobia; and (6) Abuse of psychoactive substances, such as CNS stimulants like amphetamines and hallucinogens like lysergic acid diethylamide.

Between May 2022 and November 2022, after rigorous screening based on the inclusion and exclusion criteria, 85 participants remained eligible for the study. LD was defined as an individual being initially diagnosed with HIV infection and having a CD4^+^ T cell count below 350 cells/μL or experiencing an AIDS-defining event regardless of CD4^+^ T cell count, excluding those with evidence of recent HIV infection (9, 36). Participants were categorized into the LD group and the non-LD group based on these criteria. The assessment of all participants involved a comprehensive clinical, laboratory (hematology) analysis, neuropsychiatric condition, and neuroimaging evaluation (**Supplementary Figure 1**).

### 2.2 Clinical assessments

#### 2.2.1 Diagnosis of neuropsychiatric disorders

Psychiatric diagnoses were determined by a psychiatrist using the diagnostic criteria established in the DSM-5 (35).

#### 2.2.2 Neurocognitive and sleep assessments

Neurocognitive function was assessed using the Montreal Cognitive Assessment (MoCA) (37). Sleep quality was evaluated by the Pittsburgh Sleep Quality Index (PSQI) (38).

#### 2.2.3 Other assessments

Childhood maltreatment history was evaluated utilizing the Childhood Trauma Questionnaire (CTQ) (39).

### 2.3 MRI data acquisition

Imaging data were obtained using a 1.5 T MRI scanner (Philips, Amsterdam, The Netherlands) at the Second Hospital of Beijing, with foam cushions used to minimize head movements. All subjects were instructed to lie in a relaxed position, close their eyes, and refrain from thinking about specific things without falling asleep. The detailed parameters for the whole-brain three-dimensional T1-weighted images (T1WI) and resting-state functional MRI (rs-fMRI) are provided in the supplementary material.

### 2.4 Image preprocessing

Image preprocessing and statistical analyses were performed by MATLAB R2023a (The MathWorks, Natick, MA, USA), adhering to rigorous standards. The raw image data were initially examined for imaging artifacts and anatomical anomalies. All image data acquired in digital imaging and communications in medicine format was transformed to neuroimaging informatics technology initiative format for subsequent image processing and analysis.

T1WI underwent preprocessing utilizing computational anatomy toolbox 12 (CAT12, http://dbm.neuro.uni-jena.de/cat) within Statistical Parametric Mapping 12 (SPM12, Wellcome Department of Imaging Neuroscience, UCL, UK, https://www.fil.ion.ucl.ac.uk/spm/software/spm12/) in MATLAB R2023a for voxel-based morphometry (VBM) analysis. The whole-brain T1WI were segmented into gray matter, white matter, and cerebrospinal fluid (CSF) images, which were then normalized to account for volume differences. Ultimately, all segmented images were smoothed with an 8 mm full width at half-maximum (FWHM) Gaussian kernel to enhance the signal-to-noise ratio.

The rs-fMRI data underwent preprocessing utilizing SPM12 and the REST Data Analysis Kit (REST, http://www.restfmri.net) (40). The processing pipeline included the following steps: removal of initial volumes (n = 5) to allow magnetization stabilization, slice timing correction, and motion correction. The functional images were subsequently co-registered with individual T1WI and spatially normalized utilizing the Montreal Neurological Institute template. The normalized images were resampled to an isotropic voxel size of 3.0 × 3.0 × 3.0 mm and smoothed with a 6 mm FWHM Gaussian kernel before removing linear drift. The Friston 24-parameter head motion model was employed to regress out the effects of head motion (41, 42). Then, to further mitigate nuisance signals, the mean white matter and CSF signals were regressed out. A temporal band-pass filter (0.01-0.08 Hz) was applied to filter high-frequency physiological noise and low-frequency drifts.

Ten subjects were removed from further analysis due to significant head motion (up to 2.0^°^ rotation and 2.0 mm displacement).

### 2.5 MRI data analysis

#### 2.5.1 Computation of structural data

VBM analysis was performed on the structural images. Initially, each normalized bias-corrected volume was visually inspected to identify and eliminate any volumes with artifacts and suboptimal orientations. Additionally, modulated normalized gray matter segments were examined to detect outliers and ensure sample homogeneity. We considered it qualified when the weighted average of the image and preprocessing quality was equal to or greater than 80%.

Three subjects were removed from further data analysis due to the poor quality of their structural images.

#### 2.5.2 Computation of the amplitude of low-frequency fluctuations (ALFF) and fractional ALFF maps

The ALFF value for every voxel was derived by calculating the square root of the average power spectrum within a certain frequency range (0.01-0.08 Hz). The fALFF measures the ratio of the power spectrum within a certain frequency range to that across all frequencies combined. To standardize variability across participants, the ALFF and fALFF maps of each subject were normalized using the mean ALFF and fALFF maps, respectively, for group comparison.

#### 2.5.3 Computation of the ReHo maps

The ReHo value for each voxel was computed by Kendall’s coefficient of concordance for that voxel and its 26 neighboring voxels (43). Subsequently, Gaussian smoothing with a FWHM of 6 mm was applied to ReHo maps to diminish residual differences and noise in gyral anatomy. To mitigate the global effects of inter-participant variability, we computed every subject’s mean ReHo for group comparison.

#### 2.5.4 Seed-based whole-brain functional connectivity (FC) analysis

We targeted brain regions that exhibited group differences in VBM, ALFF, fALFF, and ReHo analyses as seed regions for further FC analysis. The masks for these seed regions were derived from the automated anatomical labeling atlas (44). A Fisher *r*-to-*z* conversion was conducted to enhance the normality of FC maps, which were subsequently utilized for group comparison.

#### 2.5.5 Specific imaging statistical methods

A two-sample *t*-test was employed in voxel-based comparisons to examine the differences in VBM, ALFF, fALFF, ReHo, and FC between groups. To account for potential confounders, four covariates were included: age, years of education, CD8^+^ T cell counts at HIV diagnosis, and viral load levels at HIV diagnosis. Total intracranial volume was also incorporated as a covariate for the morphometric analysis. The significance levels were determined with a voxel threshold of *P* < 0.001. Corrections for multiple comparisons were applied utilizing the AlphaSim methods. For further exploratory analyses in conditional group comparisons, a threshold of *P* < 0.001 was applied (uncorrected for multiple comparisons). The normalized segmented gray matter images were converted into binary masks and used as statistical masks for analysis. The imaging findings were displayed using MRIcroGL software (https://www.nitrc.org/projects/mricrogl/).

### 2.6 Enzyme-linked immunosorbent assay

Endocrine hormones and neurotrophins were correlated using ELISA kits. Seven hormones and neurotrophic factors were primarily analyzed, and all assays were performed according to the manufacturers’ protocols (**Supplementary Table 1**).

### 2.7 Mass cytometry and data analysis

To investigate systemic immunity in participants with LD, mass spectrometry cell counts were conducted on peripheral blood T cell samples. A total of 23 custom-made antibodies were employed to differentiate various immune cells. **Supplementary Table 2** contains comprehensive details about the monoclonal antibodies that have been labeled with heavy metal isotopes. These pre-conjugated antibodies were purchased from Fluidigm (South San Francisco, USA). The cell labeling process followed previously established protocols (45).

In summary, extracted peripheral blood T cell samples were washed and stained with cisplatin-195Pt (Fluidigm, 201064) to exclude dead cells. Antibody staining was preceded by Fc receptor blocking using human TruStain FcX. All antibodies were used according to the manufacturer’s recommendations. After that, cell samples were subsequently washed and incubated with cell surface antibodies at low temperatures for 30 minutes. The antibody-labeled samples were then washed and incubated in 125 nM Cell-ID Intercalator-Ir (Fluidigm, USA) and diluted in phosphate-buffered saline (Sigma-Aldrich, USA) before storage at 4°C. The samples were resuspended in double-distilled water containing EQ beads (Fluidigm, South San Francisco, USA) at a concentration of 5.5 × 10^5^ cells/ml. Finally, the pre-processed samples were analyzed by the CyTOF2 mass cytometry system (Fluidigm, South San Francisco, USA).

Using a doublet-filtering approach, the raw data of each pre-processed sample were de-barcoded utilizing distinct mass-tagged barcodes. The.fcs files produced by various batches were standardized using the bead normalization technique. Subsequently, meticulous gating was performed using FlowJo software (version 10.9.0) to remove debris and dead cells. After manually gating to remove components such as beads, dimers, cell debris, and dead cells, we isolated CD45^+^ CD3^+^ T lymphocytes for subsequent analysis (**Supplementary Figure 2**). Lymphocytes were then artificially gated for further analysis in the R language. The PhenoGragh clustering technique was used to separate the cells into many clusters according to the expression levels of surface markers. To reduce dimensionality and visualize the high-dimensional data, a visual dimensionality reduction approach called *t*-distributed stochastic neighbor embedding was used. The distribution of each cluster, marker expression, and differences between groups or sample types were analyzed using R software (version 3.6.0).

### 2.8 Statistical analysis

Statistical analysis was conducted utilizing SPSS software (version 25.0; IBM Corp., Armonk, New York, USA). The significance threshold α was set at 0.05. The normal distribution of continuous data was evaluated utilizing the Shapiro-Wilk and Kolmogorov-Smirnov tests. For normally distributed data, continuous data were expressed as mean with standard deviation; for non-normally distributed data, they were expressed as medians with interquartile ranges. Based on the results of the normality tests, two-sample *t*-tests or Mann-Whitney *U*-tests were employed to compare numerical variables between groups. Categorical data were presented as a ratio. The chi-square test and Fisher’s exact test were employed to compare categorical variables between groups. For correlation analysis, we applied Pearson’s correlation analysis for normally distributed data, while Spearman’s correlation analysis for non-normally distributed data. The results of intergroup comparisons for neuroimaging and immunological variables were adjusted for four covariates: age, years of education, CD8^+^ T cell counts at HIV diagnosis, and viral load levels at HIV diagnosis. Generate box plots of intergroup comparison results using R software.

Neuropsychiatric disorders may affect clinical neuroimaging measurements in specific brain regions. Neuropsychiatric specialists made diagnoses based on DSM-5 criteria, identifying 19 related neuropsychiatric issues, including depressive disorders, bipolar disorder, anxious disorders, sleep disorder, obsessive-compulsive disorder, and alcohol-induced mental and behavioral disorders. Participants exhibiting any of these disorders were categorized as the neuropsychiatric disorder group, while those without any were categorized as the non-neuropsychiatric disorder group. A two-way factorial analysis of variance (ANOVA) was conducted to examine whether imaging changes in the brain were associated with the main effects of groups (LD vs. non-LD), neuropsychiatric disorders status (positive vs. negative), or the interaction between them.

The average time series of voxels in seed regions was extracted for every subject. The imaging results were correlated with clinical variables, neurotrophic factors, and endocrinological and immunological indicators of the participants. GraphPad Prism software (version 9.5.1; San Diego, CA, USA) was utilized to generate a diagram illustrating the statistical results.

## 3 Results

### 3.1 Characteristics of study participants

A total of 72 participants successfully concluded the study. Among them, 38 participants (52.78%) were included in the LD group, while the remaining 34 participants (47.22%) were included in the non-LD group. Compared to participants in the non-LD group, those in the LD group exhibited lower CD4^+^ T cell counts at HIV diagnosis (*P* < 0.001), lower CD4^+^ T cell counts at the initiation of ART (*P* < 0.001), lower current CD4^+^ T cell counts (*P* < 0.001), and higher viral load levels at HIV diagnosis (*P* = 0.004). No differences were observed between the two groups in terms of PSQI, CTQ, MoCA, or medication status. The demographics and clinical assessment characteristics are comprehensively outlined in **Table 1**.

**Table 1:** Demographic and clinical characteristics of all participants.

Demographic and clinical data	LD group (N = 38)	Non-LD group (N = 34)	Statistic	*P* value
Age (years)	36.00 (28.75 - 40.00)	32.00 (27.00 - 35.25)	*Z* = -1.768	0.077^a^
Height (m)	1.75 ± 0.05	1.75 ± 0.05	*t* = 0.278	0.782^b^
Weight (kg)	67.50 ± 9.20	69.62 ± 9.65	*t* = -0.953	0.344^b^
BMI (kg/m²)	21.51 (20.05 - 23.39)	22.86 (20.93 - 23.66)	*Z* = -1.568	0.117^a^
Education (years)	16.00 (15.00 - 17.00)	16.00 (12.00 - 16.00)	*Z* = -0.881	0.378^a^
Period of diagnosed HIV infection				
CD4 at HIV diagnosis (cells/μL)	252.42 (117.75 - 324.21)	482.50 (385.00 - 538.75)	*Z* = -7.287	<0.001^a^
CD8 at HIV diagnosis (cells/μL)	860.42 (724.50 - 1045.25)	1039.71 (932.64 - 1273.50)	*Z* = -3.345	0.001^a^
CD4/CD8 ratio at HIV diagnosis	0.26 (0.13 - 0.36)	0.43 (0.36 - 0.55)	*Z* = -5.234	<0.001^a^
VL at HIV diagnosis (log10 copies/mL)	4.26 (3.81 - 5.11)	3.87 (3.39 - 4.26)	*Z* = -2.883	0.004^a^
Period of initial ART start				
CD4 at initiation of ART (cells/μL)	252.42 (135.56 - 326.38)	490.81 (387.21 - 584.64)	*Z* = -6.689	<0.001^a^
CD8 at initiation of ART (cells/μL)	874.00 (714.21 - 1045.25)	1127.48 (932.75 - 1325.45)	*Z* = -3.395	0.001^a^
CD4/CD8 ratio at initiation of ART	0.26 (0.14 - 0.38)	0.43 (0.35 - 0.56)	*Z* = -4.636	<0.001^a^
VL at initiation of ART (log10 copies/mL)	4.30 ± 0.98	3.91 ± 0.63	*t* = 2.021	0.047^b^
ART regimen at initiation (INSTI/Non-INSTI - based regimen)	8/30	4/30	*χ2 =* 1.115	0.291^c^
Period of clinical and MRI assessment				
Current CD4 (cells/μL)	473.43 (341.25 - 577.25)	768.82 (569.50 - 980.00)	*Z* = -4.743	<0.001^a^
Current CD8 (cells/μL)	757.00 (536.50 - 1105.75)	965.50 (712.50 - 1226.00)	*Z* = -1.545	0.122^a^
Current CD4/CD8 ratio	0.63 ± 0.34	0.82 ± 0.25	*t* = -2.742	0.008^b^
Current virus not detectable (yes/no)	38/0	34/0	NA	NA
Current ART regimen (INSTI/Non-INSTI - based regimen)	23/15	23/11	*χ2 =* 0.394	0.530^c^
Duration between diagnosis and initiation of ART (months)	0.50 (0.40 - 5.58)	0.50 (0.38 - 0.88)	*Z* = -0.716	0.474^a^
Duration of ART (months)	68.52 ± 47.99	68.73 ± 37.53	*t* = -0.021	0.984^b^
Duration of HIV diagnosis (months)	75.00 ± 51.82	72.55 ± 39.06	*t* = 0.225	0.823^b^
Diagnosis of neuropsychiatric disorders (positive/negative)	13/25	19/15	*χ2 =* 3.413	0.065^c^
PSQI	4.00 (2.75 - 8.00)	6.50 (3.75 - 9.00)	*Z* = -1.835	0.066^a^
CTQ	60.00 (53.00 - 64.00)	61.00 (53.50 - 62.25)	*Z* = -0.198	0.843^a^
MoCA	26.00 (25.00 - 28.00)	27.50 (24.75 - 28.00)	*Z* = -0.825	0.409^a^

The continuous data were expressed as mean ± standard deviation or median (interquartile range) and the categorical data were expressed as numbers. Two-sample *t*-tests were used for continuous data with a normal distribution, while Mann-Whitney *U*-tests were used for continuous data that did not obey a normal distribution. Chi-square and Fisher’s exact tests were used to compare categorical variables. ^a^Mann-Whitney *U*-test, ^b^two-sample *t*-test, ^c^chi-square test.

LD, late HIV diagnosis; NA, not available; BMI, body mass index; CD4, CD4^+^ T cell count; CD8, CD8^+^ T cell count; VL, viral load; ART, antiretroviral therapy; INSTI, integrase strand transfer inhibitor; MRI, magnetic resonance imaging; PSQI, Pittsburgh sleep quality index; CTQ, childhood trauma questionnaire; MoCA, Montreal cognitive assessment.

Out of all participants, 13 (34.21%) from the LD group and 19 (55.88%) from the non-LD group were diagnosed with neuropsychiatric disorders, with depressive disorder being most commonly reported among them (see **Table 1**, **Supplementary Table 3** for details). No significant difference was observed in terms of neuropsychiatric condition diagnoses between the two groups (**Supplementary Table 3**).

### 3.2 Comparison of brain MRI metrics

#### 3.2.1 Brain volumetrics

**Figure 1:**
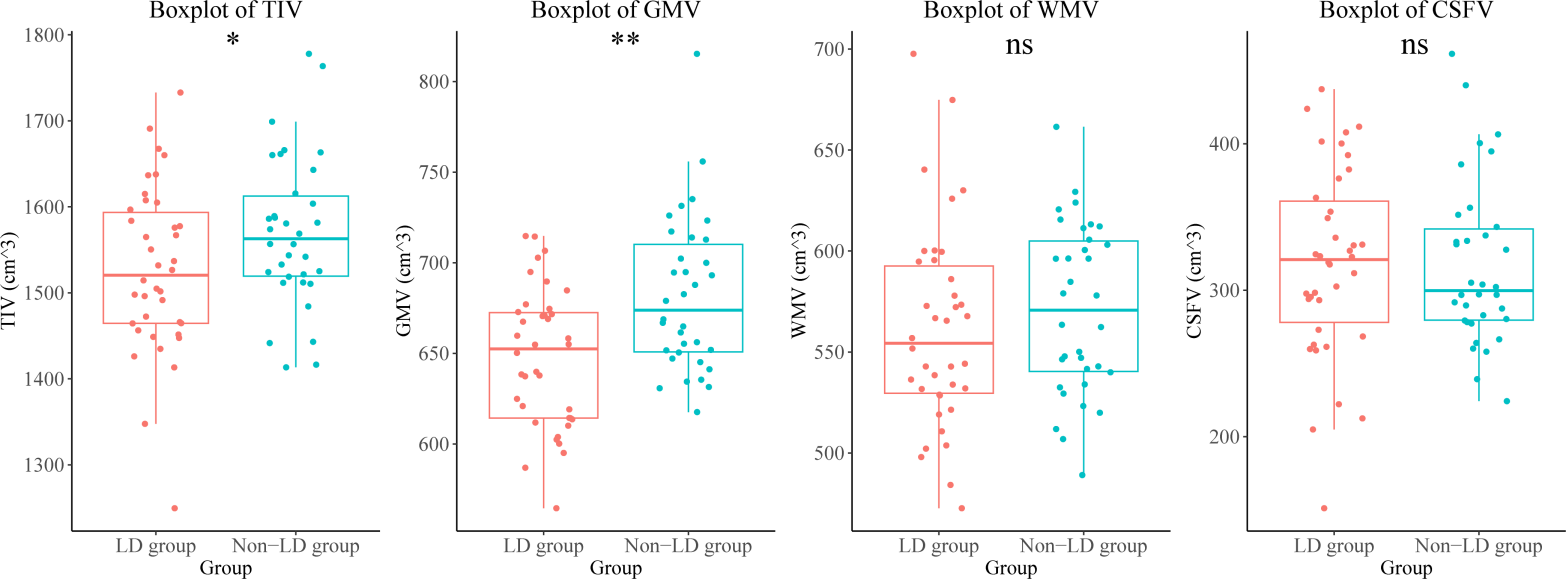
Brain volumetric differences between the LD and non-LD groups of participants. **means *P* < 0.01, *means *P* < 0.05. LD, late HIV diagnosis; TIV, total intracranial volume; GMV, gray matter volume; WMV, white matter volume; CSFV, cerebrospinal fluid volume; ns, not significant.

Participants in the LD group showed a lower total intracranial volume and total GMV than those in the non-LD group. There were no differences in the white matter volume, or CSF volume between the two groups of participants (**Figure 1**, **Supplementary Table 4**).

#### 3.2.2 GMV

**Figure 2:**
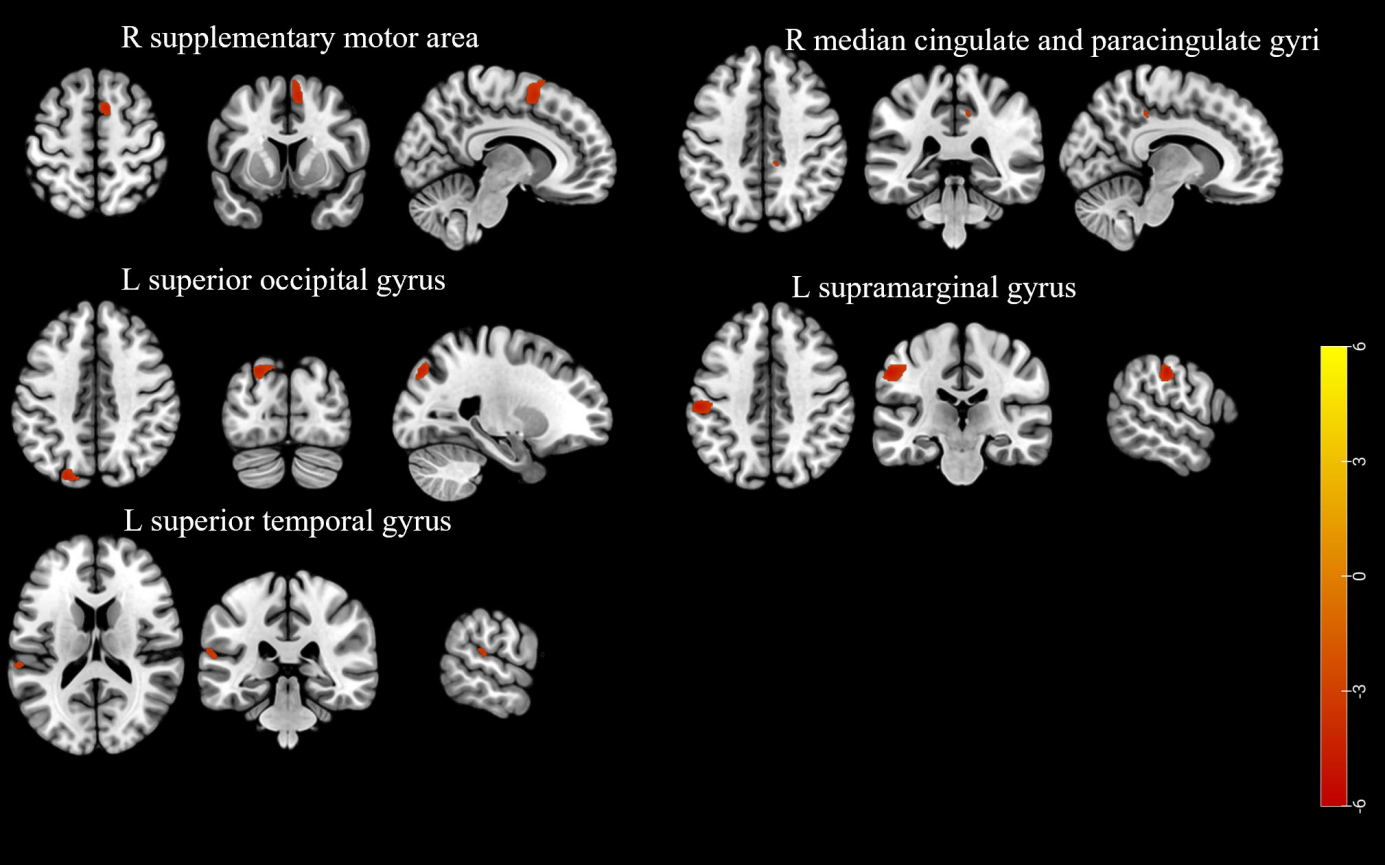
The brain region with a lower gray matter volume in the participants of the LD group compared to the non-LD group (voxel-level uncorrected *P* < 0.001). LD, late HIV diagnosis; L, left; R, right. The color bars indicate T-statistics (red/yellow).

Participants in the LD group exhibited lower GMV in the right supplementary motor area, right median cingulate and paracingulate gyri, left superior occipital gyrus, left supramarginal gyrus, and left superior temporal gyrus compared to those in the non-LD group (voxel-level uncorrected *P* < 0.001; see **Figure 2**, **Supplementary Table 5** for details).

#### 3.2.3 ALFF, fALFF, and ReHo

**Figure 3:**
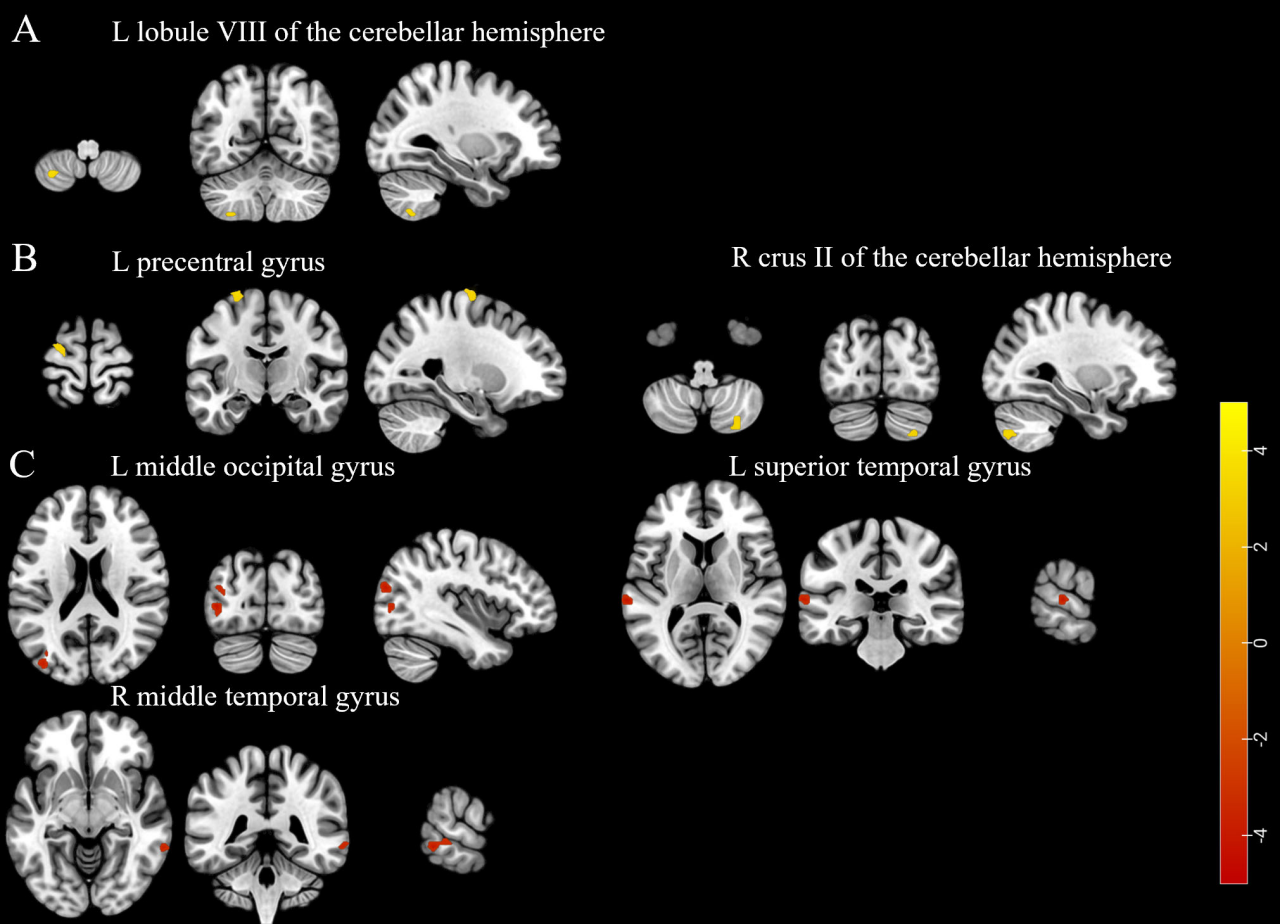
**(A)** The brain region with a higher amplitude of low-frequency fluctuations was found in the participants of the LD group compared to the non-LD group. **(B)** Brain regions exhibited higher regional homogeneity in the participants of the LD group compared to the non-LD group. **(C)** Brain regions showed lower regional homogeneity in the participants of the LD group compared to the non-LD group (voxel-level uncorrected *P* < 0.001). LD, late HIV diagnosis; L, left; R, right. The color bars indicate T-statistics (red/yellow).

Participants in the LD group had higher ALFF in the left lobule VIII of the cerebellar hemisphere, higher ReHo in the left precentral gyrus and right crus II of the cerebellar hemisphere, and lower ReHo in the left middle occipital gyrus, left superior temporal gyrus, and right middle temporal gyrus compared to those in the non-LD group (voxel-level uncorrected *P* < 0.001; see **Figure 3**, **Supplementary Tables 6**, **7** for details). However, there was no difference in the comparison of fALFF between the two groups of participants.

#### 3.2.4 FC

Compared with participants in the non-LD group, those in the LD group exhibited lower voxel-wise FC for the seed region in the left middle occipital gyrus with clusters in the right triangular part of the inferior frontal gyrus (as FC1), left superior occipital gyrus with clusters in the right inferior occipital gyrus (as FC2), and right median cingulate and paracingulate gyri with clusters in the right orbital part of the superior frontal gyrus (as FC3) (voxel-level uncorrected *P* < 0.001; see **Supplementary Tables 8**, **9** for details).

### 3.3 Intergroup comparison of endocrine markers and neurotrophic factors

**Figure 4:**
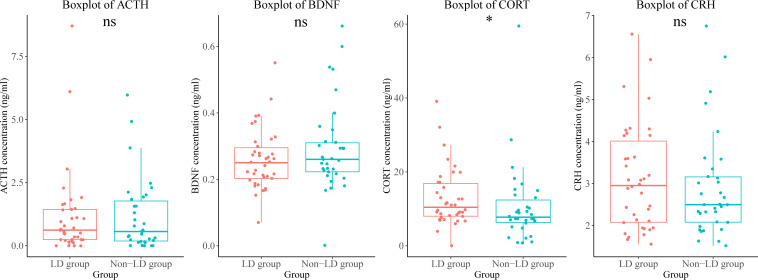
Differences in endocrine hormone and neurotrophic factor levels between the LD and non-LD groups of participants. *means *P* < 0.05. LD, late HIV diagnosis; ACTH, adrenocorticotropic hormone; BDNF, brain-derived neurotrophic factor; CORT, cortisol; CRH, corticotropin-releasing hormone; ns, not significant.

We found that plasma levels of cortisol were higher in the LD group than in the non-LD group (**Figure 4**).

### 3.4 Reduction in the number of central memory T cells and activation of double negative T cell function in the LD group

**Figure 5:**
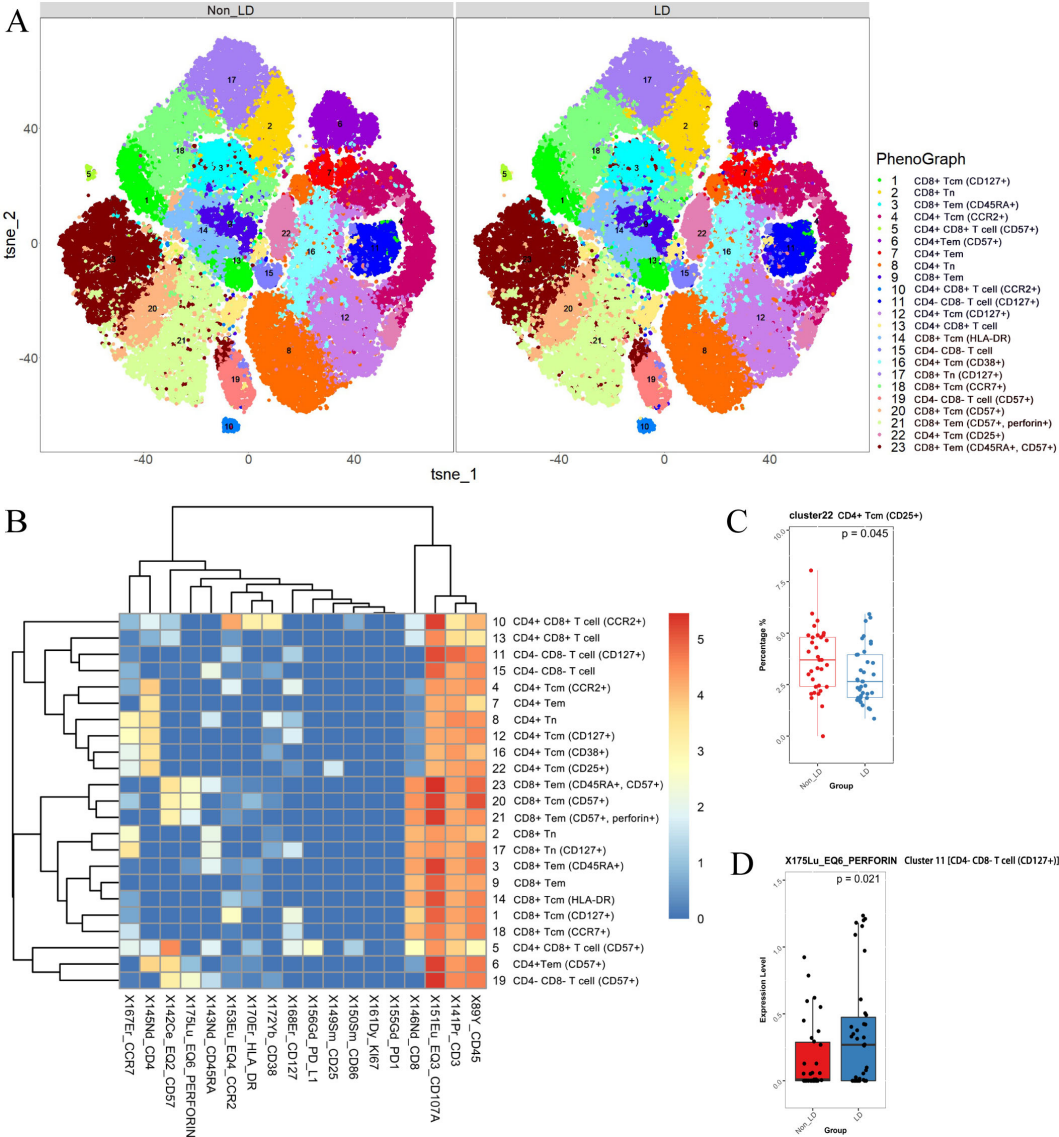
CD3^+^ T cells were analyzed and categorized into 23 clusters. **(A)** *t*-SNE plots of CD3^+^ T cells in non-LD and LD groups. **(B)** Heatmap of markers expression for 23 CD3^+^ T cell clusters. Among the 23 clusters of CD3^+^ T cells, C8 represented CD4^+^ naïve T cells (T_N_) (CD3^+^ CD4^+^ CD8^-^ CD45RA^+^ CCR7^+^); C4, C12, C16, and C22 represented CD4^+^ central memory T cells (T_CM_) (CD3^+^ CD4^+^ CD8^-^ CD45RA^-^ CCR7^+^); C6, C7 represented CD4^+^ effector memory T cells (T_EM_) (CD3^+^ CD4^+^ CD8^-^ CD45RA^-^ CCR7^-^). While C2, C17 represented CD8^+^ T_N_ (CD3^+^ CD4^-^ CD8^+^ CD45RA^+^ CCR7^+^); C1, C14, C18, and C20 represented CD8^+^ T_CM_ (CD3^+^ CD4^-^ CD8^+^ CD45RA^-^ CCR7^+^); C9, C21 represented CD8^+^ T_EM_ (CD3^+^ CD4^-^ CD8^+^ CD45RA^-^ CCR7^-^); C3, C23 represented CD8^+^ CD45RA^+^ effector memory T cells. **(C)** Different frequencies of C22 (CD4^+^ T_CM_) between non-LD and LD groups. **(D)** Expression level of perforin (in C11) between non-LD and LD groups. LD, late HIV diagnosis; *t*-SNE, *t*-distributed stochastic neighbor embedding. A *P* value < 0.05 was considered statistically significant.

The distribution of each cluster in the non-LD group and LD group, as well as the marker expression, is depicted in **Figure 5A**. Specifically, immune cells were divided into 7 categories, which were eventually divided into 23 subgroups due to the different expression of functional markers (**Figures 5A, B**). Since we failed to find obvious concentrated distribution characteristics of each marker in different clusters (**Supplementary Figure 3**), we decided to further analyze the differences in cell number and cell function of each cluster between the two groups. (**Figure 5B**).

As expected, we found a decrease in the count of C22 (CD4^+^ T_CM_) in the LD group (uncorrected *P* = 0.045, **Figure 5C**). However, we did not detect significant differences in the frequencies of other CD3^+^ T cell subpopulations between the non-LD and LD groups (data not shown). To further explore the effect of late diagnosis on immune cell function, we analyzed marker expression (CD38, CD57, human leukocyte antigen-DR, PD-1, PD-L1, CD107a, and perforin) in different subpopulations between non-LD and LD. The results showed that the LD group expressed elevated levels of perforin (in C11, which represents the double-negative T cells (DNT)) compared to the non-LD group (**Figure 5D**). The results remained significant after adding covariates (**Supplementary Table 4**).

### 3.5 Analysis of the main effects and interactions between groups and neuropsychiatric disorders on image, hormone, and immune results

The main effects of LD were observed in the total GMV and all brain regions with differences in VBM analysis. The group differences in ReHo were found in the left precentral gyrus, which exhibited a main effect of neuropsychiatric disorders. The ALFF result in the left lobule VIII of the cerebellar hemisphere and the VBM result in the left supramarginal gyrus showed the interaction between LD and neuropsychiatric disorders.

For the intergroup analysis results of endocrine and immune differences, we found a main effect of LD on perforin expression in cluster 11 (CD4^-^ CD8^-^ T cells), a main effect of neuropsychiatric disorders on the frequency of cluster 22 (CD4^+^ T_CM_), and an interaction effect between group and neuropsychiatric disorders on cortisol levels (see **Supplementary Table 10** for details).

Additional analysis of metrics showing interaction effects revealed that, for ALFF results in the left lobule VIII of the cerebellar hemisphere, the LD group exhibited significantly higher ALFF values than the non-LD group only when neuropsychiatric disorders were present (mean difference = -0.108, *P* = 0.002), while there was no difference between the two groups without neuropsychiatric disorders (*P* = 0.756). In the LD group, neuropsychiatric disorder status was significantly correlated with the increased ALFF values (mean difference = -0.083, *P* = 0.011), while no significant changes in ALFF values were observed in the non-LD group with neuropsychiatric disorders (*P* = 0.626). For VBM analysis results in the left supramarginal gyrus, only when neuropsychiatric disorders were present, the non-LD group showed significantly higher GMV than the LD group (mean difference = 0.052, *P* < 0.001), whereas there was no difference between the two groups without neuropsychiatric disorders (*P* = 0.165). In both non-LD and LD groups, the GMV difference between individuals with and without neuropsychiatric disorders was not significant. For cortisol levels, only in the presence of neuropsychiatric disorders did the LD group have significantly higher cortisol levels than the non-LD group (mean difference = -7.981 ng/mL, *P* = 0.017), whereas there was no difference between the two groups without neuropsychiatric disorders (*P* = 0.483). In both the non-LD and LD groups, the cortisol levels did not differ significantly between individuals with and without neuropsychiatric disorders (**Supplementary Table 11**).

### 3.6 Correlations of the imaging alterations with neurotrophic factors, endocrinological and immunological indicators, and clinical characteristics

**Figure 6:**
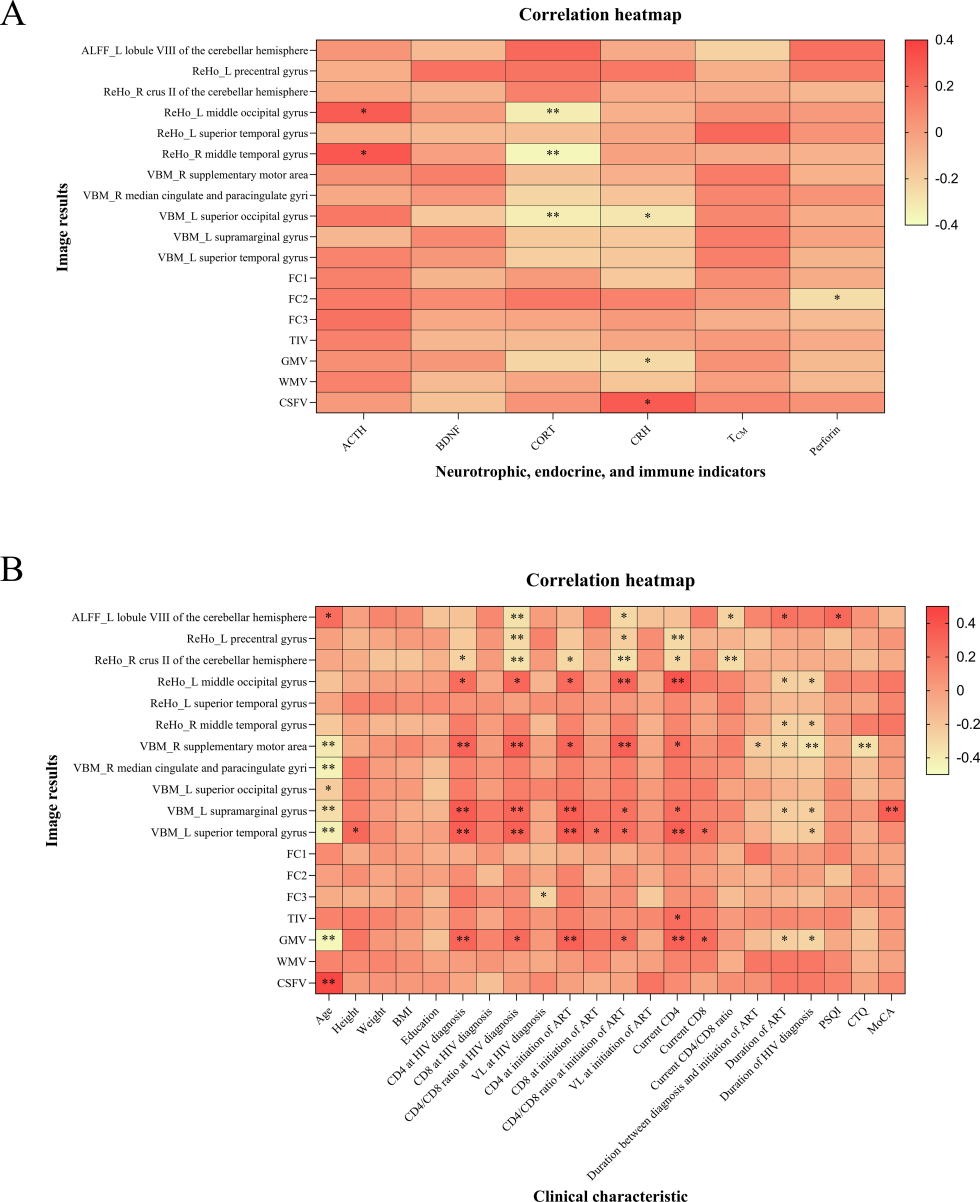
Correlation analysis results of all participants. **(A)** The imaging alterations were correlated with neurotrophic factors, endocrinological and immunological indicators. **(B)** The correlation analysis of imaging alterations with clinical characteristics. FC1, FC2, and FC3 represented the voxel-wise FC for the seed region in the left middle occipital gyrus with clusters in the right triangular part of the inferior frontal gyrus, the voxel-wise FC for the seed region in the left superior occipital gyrus with clusters in the right inferior occipital gyrus, and the voxel-wise FC for the seed region in the right median cingulate and paracingulate gyri with clusters in the right orbital part of the superior frontal gyrus, respectively. ALFF, amplitude of low-frequency fluctuation; ReHo, regional homogeneity; VBM, voxel-based morphometry; FC, functional connectivity; TIV, total intracranial volume; GM, gray matter; WM, white matter; CSF, cerebrospinal fluid; L, left; R, right; ACTH, adrenocorticotropic hormone; BDNF, brain-derived neurotrophic factor; CORT, cortisol; CRH, corticotropin-releasing hormone; T_CM_, central memory T cells; BMI, body mass index; CD4, CD4^+^ T cell count; CD8, CD8^+^ T cell count; VL, viral load; ART, antiretroviral therapy; PSQI, Pittsburgh sleep quality index; CTQ, childhood trauma questionnaire; MoCA, Montreal cognitive assessment. ^*^ *P* < 0.05, ^**^ *P* < 0.01. The color bar is used to represent the correlation coefficients in the heatmap.

Among all participants, we conducted two correlation analyses. The imaging alterations were correlated with the levels of neurotrophic factors, endocrinological indicators, and immunological markers. The plasma levels of cortisol were negatively correlated with ReHo values in the left middle occipital gyrus and right middle temporal gyrus, as well as GMV in the left superior occipital gyrus. Additionally, FC2 values were negatively correlated with the expression of perforin (**Figure 6A**).

The correlation analysis between imaging alterations and clinical characteristics indicated that age, duration of ART, and duration of HIV diagnosis were negatively correlated with total GMV and certain brain regions with differences in VBM analysis. Furthermore, the MoCA score was positively correlated with GMV in the left supramarginal gyrus (**Figure 6B**).

## 4 Discussion

In this study, we conducted clinical, neuroimaging, neurotrophic factors, endocrinology, and immunology comparisons between the LD and non-LD groups of participants. We discovered that compared to participants in the non-LD group, those in the LD group exhibited a lower total GMV and reductions in GMV in specific brain regions such as the left supramarginal gyrus, etc. Additionally, we observed a main effect of LD on brain gray matter atrophy, as well as differences between the two groups in terms of ALFF, ReHo, and voxel-wise FC. Furthermore, we found higher levels of cortisol and a lower frequency of T_CM_ cells, along with elevated expression levels of perforin by double-negative T cells in the LD group. A significant association was observed between gray matter atrophy, altered brain function patterns, clinical characteristics, neurotrophic factors, and endocrinological and immunological indicators. These results provide evidence that LD may potentially exacerbate brain injury as well as disrupt endocrine and immune regulation in HIV-infected MSM.

In brain structural imaging comparisons, the LD group demonstrated lower total GMV and reduced volumes in specific critical brain regions compared to the non-LD group. These observations suggest that LD may potentially contribute to HIV-related brain atrophy, particularly in gray matter regions such as the left supramarginal gyrus. The supramarginal gyrus, a component of the inferior parietal lobule involved in language perception and processing (46, 47), also contributes to the somatosensory association cortex, crucial for neural representation of motor actions. Deficits in generating mental movement representations have been associated with lesions in this area (48). Literature indicates that atrophy of the left supramarginal gyrus leads to conduction aphasia (49, 50). Our study revealed reduced GMV specifically in the left supramarginal gyrus among individuals with LD compared to those without LD. Furthermore, GMV within this region is positively correlated with MoCA scores. Therefore, these results imply that the decline in general language function and cognitive abilities in MSM with LD in the future may be primarily linked to the atrophy in this region.

In the rs-fMRI results, we observed higher ALFF within the left lobule VIII of the cerebellar hemisphere among individuals with LD. Cerebellar lobule VIII is a component of the sensorimotor cerebellum, active during somatosensory and sensorimotor tasks (51, 52). Relevant literature has shown that patients with a tremor-dominant subtype of Parkinson’s disease exhibit increased ALFF values in the right cerebellar lobule VIII compared to healthy controls, and ALFF values in the bilateral cerebellar lobule VIII positively correlate with tremor severity in Parkinson’s disease patients. Our findings indicate that elevated ALFF in the left cerebellar lobule VIII among individuals with LD may contribute to pathological activation patterns and further impair motor function in the brain. Considering the essential role of MRI in the early detection of neuropathological brain changes, further investigation is warranted to explore these findings.

Cortisol, a glucocorticoid hormone secreted by the adrenal cortex in response to stress and inflammatory responses (53–55), has been previously reported to cause damage to hippocampal neurons due to chronic elevation caused by long-term stress (56). Our study revealed higher cortisol levels in the LD group, suggesting elevated levels of stress and inflammation in MSM with LD. In the correlation analysis, we observed a significant association between plasma cortisol levels and radiographic outcomes. Lower ReHo values in the left middle occipital gyrus and right middle temporal gyrus, along with decreased GMV in the left superior occipital gyrus, were found to be correlated with higher cortisol levels. We propose that regions exhibiting lower ReHo values reflect decreased synchronization of time series in local brain regions, while decreased GMV represents impaired brain function. These findings indicate that cortisol may serve as a reliable biomarker for adverse prognosis in MSM with LD.

Among immune cells, we focused on the roles of T cells in peripheral immunity. Compared with the non-LD group, we found that the LD group exhibited a lower frequency of T_CM_ cells and higher levels of perforin expression in double-negative T cells. T_CM_ cells are intermediate memory T cells that do not display effector function and play an important role in memory immune responses (57, 58). The decline in the frequency of T_CM_ cells may lead to impaired immune memory, resulting in a loss of effective recall response and protection against previously encountered pathogens. DNT cells are unique antigen-specific regulatory T cells (59, 60), and increased expression of perforin in the LD group implies heightened cytotoxic function among DNTs in this group. Since DNT also play a role in promoting neuroinflammation (61), their active function within the LD group may have negative implications for inflammation promotion. In summary, our results suggest that individuals with LD display compromised hormone levels and immune cell profiles, potentially indicating detrimental effects on their immune system.

This study uncovers the relationship between immunity, endocrine dysfunction, and brain injury in PLWH with LD, offering new intervention targets for clinical treatment. For example, the study observed that plasma cortisol levels were negatively correlated with the ReHo values of the left middle occipital gyrus and right middle temporal gyrus, as well as the GMV of the left superior occipital gyrus. Abnormal cortisol levels are associated with brain structural and functional changes, indicating the important role of HPA axis dysfunction in HIV infection. These findings provide a biological foundation for early detection and personalized treatment in clinical settings. Based on the significant correlation between imaging and biological indicators, a three-tier intervention strategy is recommended: strengthening early screening through community rapid testing and pre-exposure prophylaxis, establishing an LD risk warning system; conducting management interventions for cortisol and peripheral immune abnormalities; and offering cognitive rehabilitation training based on MRI biomarkers for individuals with structural and functional brain changes. These multi-dimensional interventions should be integrated with existing antiretroviral therapy to create a comprehensive prevention and control system for the adverse effects associated with LD.

Due to various factors, individuals with HIV are susceptible to comorbid neuropsychiatric disorders, which may influence neuroimaging results associated with HIV. This study revealed the interaction between LD and neuropsychiatric disorders in the HIV-positive MSM population regarding brain structural and functional changes, as well as peripheral endocrine levels, through two-way ANOVA and simple effects testing. The interaction analysis indicates that LD and neuropsychiatric disorders synergistically cause a significant increase in ALFF values in the left lobule VIII of the cerebellar hemisphere, but only in the LD group; neuropsychiatric disorders were significantly correlated with the increase in ALFF values, while no similar trend was observed in the non-LD group; only in the presence of neuropsychiatric disorders, the non-LD group had significantly higher GMV in the left supramarginal gyrus compared to the LD group; also only in the presence of neuropsychiatric disorders, the LD group showed significantly higher cortisol levels than the non-LD group. These results suggest that the effect of LD on brain structure and function is not independent but rather synergistically affected by neuropsychiatric disorders. For example, the increase in ALFF may reflect compensatory enhancement or abnormal synchronization of neuronal activity in specific brain regions in those diagnosed later, but the exact pathophysiological mechanism needs to be further validated with multimodal data.

Our study has prominent strengths. First, this is the first study applying multimodal MRI to assess brain structure and function in PLWH with a late diagnosis. Second, by integrating multiple technologies, interdisciplinary methods were employed to further investigate associations between clinical, imaging, endocrine, and immune aspects. Furthermore, some previous clinical studies using psychiatric screening measures such as the general health questionnaire may have introduced bias, leading to an overestimation or underestimation of psychiatric disorders. In the present study, expert psychiatrists diagnosed comprehensive psychiatric disorders using standard diagnostic criteria to ensure the accuracy of this investigation. Previous studies have mainly focused on the general changes in brain structure and function in PLWH or the effects of HIV on the immune and endocrine systems. For instance, Sui et al. explored the impact of HIV infection on the cerebral cortex, identifying brain GMV atrophy and abnormal activity in the fronto-parietal and occipital networks in PLWH (8). However, these studies have not sufficiently accounted for the role of the timing of diagnosis, nor have they systematically evaluated the combined effects of LD on the immune, neurological, and endocrine systems, especially within the high-risk MSM population. This study is the first to examine the comprehensive impact of LD on the neurological system, immune system, and endocrine factors in MSM, revealing the association between LD and more severe immune dysfunction, neurodegenerative changes, and endocrine disturbances, providing new research data and insights for the biological mechanisms and clinical interventions of HIV infection.

However, our study also has limitations. First, this is a cross-sectional research project lacking longitudinal analysis of changes in brain structure and function, which might overlook valuable information. Second, only HIV-positive MSM were included in this study, which may not fully represent the entire HIV-infected population. It is suggested that future studies explore longitudinal designs to reveal the causal relationships between immune and endocrine dysregulation and brain injury, and enhance the external validity of the study by incorporating diverse populations (such as PLWH from different genders, races, and socioeconomic backgrounds). Third, the study used 1.5 T MRI, which has a lower overall signal-to-noise ratio and spatial resolution, potentially leading to reduced sensitivity. With the use of 3 T and 7 T MRI technology, future research will achieve significant progress in improving spatial resolution and signal-to-noise ratio, revealing the microscopic changes in HIV-related brain injury. Fourth, the study only collected peripheral blood for the analysis of neurotrophic factors, endocrine, and immune markers, lacking cerebrospinal fluid testing and the evaluation of other types of biomarkers, such as neurodegenerative markers.

In summary, our study demonstrates that LD in MSM is associated with gray matter atrophy, functional abnormalities, endocrine disruption, and immune dysregulation. Further analysis reveals an association between brain injury and both endocrine and immune abnormalities. This study emphasizes the potential adverse effects of LD on the long-term prognosis of MSM and underscores the clinical importance of early diagnosis. Ultimately, we believe that this research contributes to both the theoretical foundation and data support for controlling the LD epidemic.

## Data Availability

The raw data supporting the conclusions of this article will be made available by the authors, without undue reservation.
