# Supplementary material for: Brain injury, endocrine disruption, and immune dysregulation in HIV-positive men who have sex with men with late HIV diagnosis
Source: Front Immunol. 2025 Mar 19;16:1436589. doi: 10.3389/fimmu.2025.1436589 (PMC11961418; doi:10.3389/fimmu.2025.1436589)
Supplement: Supplementary file 1 [file DataSheet1.docx]

Supplementary Material

**Supplementary Data**

**Methods**

**The magnetic resonance imaging (MRI) scan parameters**

For three-dimensional T1-weighted image, the parameters were as follows: shortest repetition time/echo time (TR/TE) = 8.3/3.9 ms, matrix = 256 × 227, flip angle = 12^°^, slice thickness = 1 mm, slices = 384, field of view = 256 mm × 256 mm. Resting-state functional MRI of the entire brain was acquired by a gradient echo-planar imaging sequence. Acquisition parameters were as follows: TR/TE = 4019.8/30 ms, slices = 40, flip angle = 90^°^, matrix = 64 × 62, slice thickness = 3.5 mm, no gap, and volumes = 102, scanning time = 6 min 52 s.

**Post-hoc power analysis**

Since this is an exploratory study, lacking prior studies as references, we conducted a post-hoc power analysis. The detailed process is as follows: first, we extracted key brain regions with significant between-group differences, such as the gray matter volume of the left supramarginal gyrus.

Then, we calculated the effect size (Cohen's d) using the relevant formula and performed the power calculation using G*Power software (version 3.1.9.7).

The formula for calculating Cohen’s d is as follows:

$$Cohen^{'}s d=\frac{M_{1}-M_{2}}{SD_{pooled}}$$

In this formula, *M*_1_ and *M*_2_ represent the means of the two groups, and *SD_pooled_* is the pooled standard deviation.

The formula for calculating *SD_pooled_* is as follows:

$$SD_{pooled}=\sqrt{\frac{\left( n_{1}-1 \right)SD_{1}^{2}+\left( n_{2}-1 \right)SD_{2}^{2}}{n_{1}+n_{2}-2}}$$

Where n_1_ and n_2_ are the sample sizes of the two groups, SD_1_ and SD_2_ are the standard deviations of the two groups, and the denominator (n_1_+n_2_−2) indicates the degrees of freedom.

**Results**

Data from key brain regions were extracted for each participant, including the gray matter volume of the left supramarginal gyrus. Detailed results are available in Supplementary Table 12. The mean for the LD group was M_1_ = 0.3593, and the mean for the non-LD group was M_2_ = 0.3930. The pooled standard deviation was calculated as *SD_pooled_* = 0.0367 and the effect size as *Cohen's d* = 0.917 using the appropriate formula. Finally, using G*Power software (α = 0.05), the calculated power was 0.969 (see Supplementary Figure 4).

**
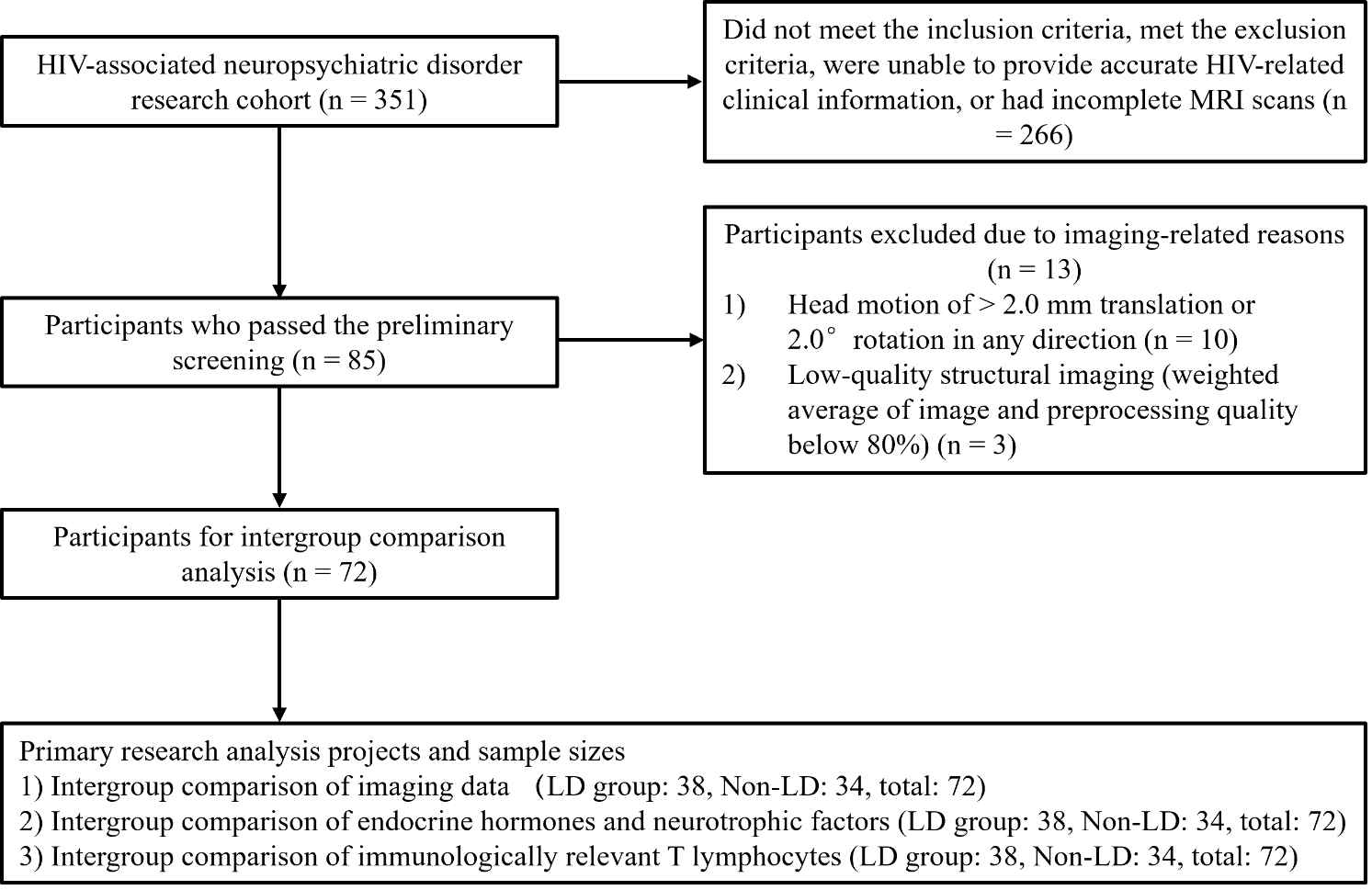
**

**Supplementary Figure 1.** Study flow.

Abbreviations: HIV, human immunodeficiency virus; MRI; magnetic resonance imaging; LD, late HIV diagnosis.

**Supplementary Table 1.** Enzyme-linked immunosorbent assay kits for hormones and neurotrophic factors.

| **Names** | **ELISA kits** |
| --- | --- |
| BDNF | Human BDNF ELISA 96T |
| Cortisol | Human Cortisol EIA 96T |
| CRH | Human CRH binding protein ELISA 96T |
| ACTH | Human Adrenocorticotropic Hormone EIA 96T |

ELISA, Enzyme-linked immunosorbent assay; BDNF, brain-derived neurotrophic factor; CRH, corticotropin-releasing hormone; ACTH, adrenocorticotropic hormone.

**Supplementary Table 2.** Antibodies and isotopes used for mass cytometry.

| **Isotopes Name** | **Antibody Name** | **Isotopes Name** | **Antibody Name** |
| --- | --- | --- | --- |
| 209Bi | CD16 | 175Lu | PERFORIN |
| 142Ce | CD57 | 143Nd | CD45RA |
| 161Dy | KI67 | 145Nd | CD4 |
| 162Dy | FOXP3 | 146Nd | CD8 |
| 167Er | CCR7 | 141Pr | CD3 |
| 168Er | CD127 | 144Sm | CD31 |
| 170Er | HLA_DR | 148Sm | CD14 |
| 151Eu | CD107A | 149Sm | CD25 |
| 153Eu | CCR2 | 150Sm | CD86 |
| 154Gd | CD163 | 89Y | CD45 |
| 155Gd | PD1 | 172Yb | CD38 |
| 156Gd | PD_L1 |  |  |

Bi, Bismuth; Ce, Cerium; Dy, Dysprosium; Er, Erbium; Eu, Europium; Gd, Gadolinium; FOXP, Forkhead Box P; CCR, C-C chemokine receptor; HLA_DR, Human Leukocyte Antigen-DR; PD, Programmed Cell Death Protein; Lu, Lutetium; Nd, Neodymium; Pr, Praseodymium; Sm, Samarium; Y, Yttrium; Yb, Ytterbium.





**Supplementary Figure 2.** Gating strategy for defining subsets of CD3^+^ cells. **(A)** Gating all cells using EQ3 and EQ4. **(B)** Gating singlets using 193lr and Event length. **(C)** Gating living cells using 195 Pt. **(D)** Gating CD45^+^ cells using CD45. **(E)** Gating CD45^+^ CD3^+^ cells using CD3.

**Supplementary Table 3.** Diagnostic results of neuropsychiatric conditions differences between the LD and non-LD groups.

| **Neuropsychiatric conditions** | **LD group** | **Non-LD group** | **Statistic** | ***P* value** |
| --- | --- | --- | --- | --- |
|  | **(N = 38)** | **(N = 34)** |  |  |
| Bipolar affective disorder (positive/negative) | 1/37 | 1/33 | NA | 1.000^a^ |
| Bipolar I disorder (positive/negative) | 1/37 | 1/33 | NA | 1.000^a^ |
| Bipolar II disorder (positive/negative) | 0/38 | 0/34 | NA | NA |
| Depressive disorder (positive/negative) | 9/29 | 12/22 | *χ2* = 1.171 | 0.279^b^ |
| Depressive symptoms (positive/negative) | 10/28 | 13/21 | *χ2* = 1.173 | 0.279^b^ |
| Suicidal symptoms (positive/negative) | 4/34 | 7/27 | *χ2* = 1.404 | 0.236^b^ |
| Major depression disorder (positive/negative) | 8/30 | 11/23 | *χ2* = 1.180 | 0.277^b^ |
| Persistent depressive disorder (positive/negative) | 3/35 | 4/30 | *χ2* = 0.024 | 0.877^b^ |
| Unspecified depressive disorder (positive/negative) | 0/38 | 1/33 | NA | 0.472^a^ |
| Anxiety disorders (positive/negative) | 5/33 | 7/27 | *χ2* = 0.713 | 0.398^b^ |
| Anxiety symptom (positive/negative) | 7/31 | 13/21 | *χ2* = 3.512 | 0.061^b^ |
| Panic disorder (positive/negative) | 3/35 | 2/32 | *χ2* < 0.001 | 1.000^b^ |
| Agoraphobia (positive/negative) | 1/37 | 1/33 | NA | 1.000^a^ |
| Social anxiety disorder (positive/negative) | 1/37 | 2/32 | *χ2* = 0.010 | 0.922^b^ |
| Generalized anxiety disorder (positive/negative) | 2/36 | 5/29 | *χ2* = 0.906 | 0.341^b^ |
| Obsessive-compulsive disorder (positive/negative) | 1/37 | 5/29 | *χ2* = 2.026 | 0.155^b^ |
| Insomnia disorder (positive/negative) | 5/33 | 7/27 | *χ2* = 0.713 | 0.398^b^ |
| Insomnia symptoms (positive/negative) | 14/24 | 17/17 | *χ2* = 1.267 | 0.260^b^ |
| Alcohol use disorder (positive/negative) | 1/37 | 2/32 | *χ2* = 0.010 | 0.922^b^ |

The categorical data were expressed as ratios. Chi-square and Fisher’s exact tests were used to compare categorical variables. ^a^Fisher’s exact test; ^b^chi-square test.

LD, late HIV diagnosis; NA, not available.**Supplementary Table 4.** Descriptive statistics and ANCOVA results for brain volume indicators and immune markers to LD and non-LD groups.

|  | **Group, adjusted mean ± SE** | |  | **ANCOVA** | | |
| --- | --- | --- | --- | --- | --- | --- |
| **Domain** | **LD group**  **(n = 38)** | **Non-LD group**  **(n = 34)** |  | ***F*** | ***P*** | **Effect size** |
| TIV | 1517.94 ± 16.28 | 1579.71 ± 17.37 |  | 5.819 | 0.019 | 0.081 |
| GMV | 649.04 ± 6.50 | 679.66 ± 6.94 |  | 8.967 | 0.004 | 0.12 |
| WMV | 561.94 ± 8.21 | 570.13 ± 8.76 |  | 0.403 | 0.528 | 0.006 |
| CSFV | 306.96 ± 9.56 | 329.91 ± 10.20 |  | 2.33 | 0.132 | 0.034 |
| Cluster 22 [CD4^+^ T_CM_ (CD25^+^)] | 2.77 ± 0.25 | 3.89 ± 0.27 |  | 7.944 | 0.006 | 0.107 |
| Perforin expression in cluster 11 [CD4^-^ CD8^-^ T cells (CD127^+^)] | 0.38 ± 0.06 | 0.14 ± 0.07 |  | 5.941 | 0.017 | 0.083 |

Means were adjusted for age, years of education, CD8 counts at HIV diagnosis, and viral load levels at HIV diagnosis. ANCOVA, analysis of covariance; SE, standard error; LD, late HIV diagnosis; TIV, total intracranial volume; GMV, gray matter volume; WMV, white matter volume; CSFV, cerebrospinal fluid volume.**Supplementary Table 5.** Gray matter volume differences between the LD and non-LD groups.

| **Brain region** | **Peak MNI coordinates** | | | **T value** | **Cluster Size** | **Effect size** |
| --- | --- | --- | --- | --- | --- | --- |
|  | **X** | **Y** | **Z** |  |  |  |
| **LD < Non-LD** |  |  |  |  |  |  |
| L supramarginal gyrus | -58 | -24 | 42 | 5.1205 | 538^*^ | -0.917 |
| R supplementary motor area | 8 | 9 | 57 | 4.4316 | 371 | -0.630 |
| L superior occipital gyrus | -24 | -82 | 42 | 4.4502 | 337 | -0.666 |
| L superior temporal gyrus | -62 | -33 | 16 | 3.7441 | 78 | -0.947 |
| R median cingulate and paracingulate gyri | 10 | -36 | 42 | 3.8282 | 24 | -0.620 |

Coordinates (X, Y, Z) refer to the peak MNI coordinates of brain regions with peak intensity (voxel-level uncorrected *P* < 0.001). ^*^Corrected for multiple comparisons (AlphaSim correction, voxel level *P* < 0.001, cluster level *P* < 0.05).

LD, late HIV diagnosis; MNI, Montreal Neurological Institute; L, left; R, right.

**Supplementary Table 6.** The amplitude of low-frequency fluctuation differences between the LD and non-LD groups.

| **Brain region** | **Peak MNI coordinates** | | | **T value** | **Cluster Size** | **Effect size** |
| --- | --- | --- | --- | --- | --- | --- |
|  | **X** | **Y** | **Z** |  |  |  |
| **LD > Non-LD** |  |  |  |  |  |  |
| L lobule VIII of the cerebellar hemisphere | -30 | -60 | -57 | 3.9069 | 11 | 0.486 |

Coordinates (X, Y, Z) refer to the peak MNI coordinates of brain regions with peak intensity (voxel-level uncorrected *P* < 0.001).

LD, late HIV diagnosis; MNI, Montreal Neurological Institute; L, left; R, right.

**Supplementary Table 7.** Regional homogeneity differences between the LD and non-LD groups.

| **Brain region** | **Peak MNI coordinates** | | | **T value** | **Cluster Size** | **Effect size** |
| --- | --- | --- | --- | --- | --- | --- |
|  | **X** | **Y** | **Z** |  |  |  |
| **LD > Non-LD** |  |  |  |  |  |  |
| L precentral gyrus | -21 | -18 | 78 | 3.8838 | 46^*^ | 0.480 |
| R crus II of the cerebellar hemisphere | 30 | -81 | -48 | 3.7002 | 18 | 0.359 |
| **LD < Non-LD** |  |  |  |  |  |  |
| L middle occipital gyrus | -39 | -84 | 21 | 4.2145 | 28 | -0.989 |
| R middle temporal gyrus | 66 | -45 | -6 | 4.2439 | 24 | -0.692 |
| L middle occipital gyrus | -42 | -81 | 6 | 4.5432 | 18 | -0.763 |
| L superior temporal gyrus | -66 | -30 | 9 | 4.2301 | 17 | -0.445 |

Coordinates (X, Y, Z) refer to the peak MNI coordinates of brain regions with peak intensity (voxel-level uncorrected *P* < 0.001).

^*^Corrected for multiple comparisons (AlphaSim correction, voxel level *P* < 0.001, cluster level *P* < 0.05).

LD, late HIV diagnosis; FDR, false discovery rate; MNI, Montreal Neurological Institute; L, left; R, right.

**Supplementary Table 8.** Seed regions definition for functional connectivity analysis.

| **Serial number** | **Seed regions** | **Source** |
| --- | --- | --- |
| Seed 1 | L lobule VIII of the cerebellar hemisphere | ALFF |
| Seed 2 | L precentral gyrus | ReHo |
| Seed 3 | R crus II of the cerebellar hemisphere | ReHo |
| Seed 4 | L middle occipital gyrus | ReHo |
| Seed 5 | R middle temporal gyrus | ReHo |
| Seed 6 | L superior temporal gyrus | ReHo, VBM |
| Seed 7 | L supramarginal gyrus | VBM |
| Seed 8 | R supplementary motor area | VBM |
| Seed 9 | L superior occipital gyrus | VBM |
| Seed 10 | R median cingulate and paracingulate gyri | VBM |

ALFF, amplitude of low-frequency fluctuation; ReHo, regional homogeneity; VBM, voxel-based morphometry; L, left; R, right.

**Supplementary Table 9.** Functional connectivity differences between the LD and non-LD groups.

| **Serial number** | **Contrast/Seed regions** | **Connected regions** | **Peak MNI coordinates** | | | **T value** | **Cluster Size** | **Effect size** |
| --- | --- | --- | --- | --- | --- | --- | --- | --- |
|  |  |  | **X** | **Y** | **Z** |  |  |  |
|  | **LD < Non-LD** |  |  |  |  |  |  |  |
| Seed 4 | L middle occipital gyrus | R triangular part of the inferior frontal gyrus | 51 | 33 | 15 | 4.2007 | 10 | -0.035 |
| Seed 9 | L superior occipital gyrus | R inferior occipital gyrus | 42 | -84 | -15 | 4.1877 | 10 | -0.176 |
| Seed 10 | R median cingulate and paracingulate gyri | R orbital part of the superior frontal gyrus | 12 | 63 | -18 | 3.8223 | 13 | -0.392 |

Coordinates (X, Y, Z) refer to the peak MNI coordinates of brain regions with peak intensity (voxel-level uncorrected *P* < 0.001).

LD, late HIV diagnosis; MRI, magnetic resonance imaging; MNI, Montreal Neurological Institute; L, left; R, right.


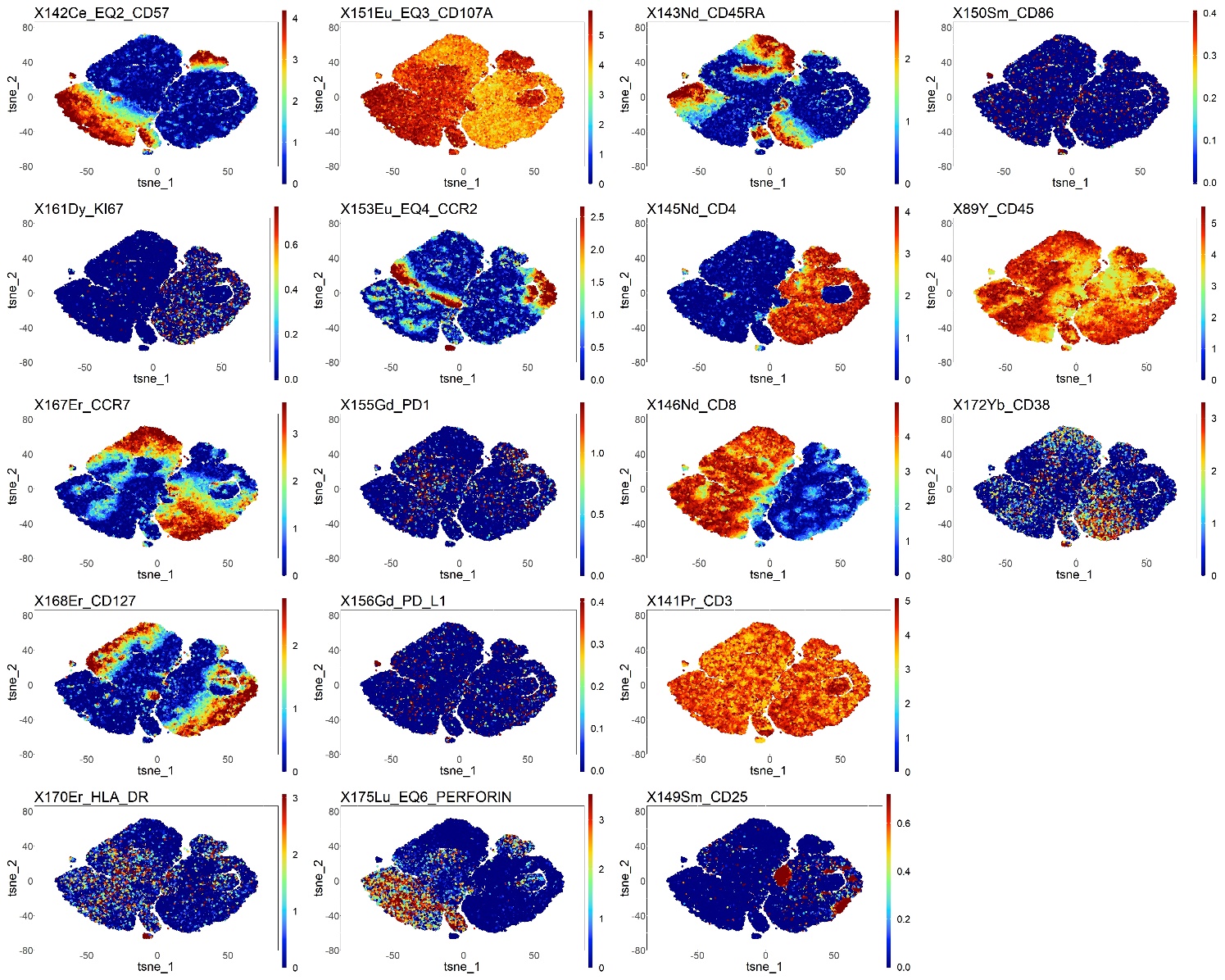


**Supplementary Figure 3.** Expression distribution of selected markers across clusters. Dark red color: high expression; dark blue color: no expression.

**Supplementary Table 10.** Main effects and interaction of groups and neuropsychiatric disorders on image, hormone and immune results.

| **Outcome indicators** | **Group** | **Negative** | **Positive** | **Main effect of groups** | | | **Main effect of neuropsychiatric disorders** | | | **Interaction of groups and neuropsychiatric disorders** | | |
| --- | --- | --- | --- | --- | --- | --- | --- | --- | --- | --- | --- | --- |
|  |  |  |  |  |  |  |  |  |  |  |  |  |
|  |  |  |  |  |  |  |  |  |  |  |  |  |
|  |  | **mean ± SD** | **mean ± SD** | ***F*** | ***P*** | **Partial η^2^** | ***F*** | ***P*** | **Partial η^2^** | ***F*** | ***P*** | **Partial η^2^** |
| **ALFF** |  |  |  |  |  |  |  |  |  |  |  |  |
| L lobule VIII of the cerebellar hemisphere | LD | 0.90 ± 0.08 | 0.98 ± 0.11 | 6.847 | 0.011 | 0.091 | 2.259 | 0.138 | 0.032 | 4.831 | 0.031 | 0.066 |
|  | Non-LD | 0.89 ± 0.10 | 0.87 ± 0.08 |  |  |  |  |  |  |  |  |  |
| **ReHo** |  |  |  |  |  |  |  |  |  |  |  |  |
| L precentral gyrus | LD | 0.81 ± 0.04 | 0.80 ± 0.05 | 2.625 | 0.110 | 0.037 | 4.887 | 0.030 | 0.067 | 0.569 | 0.453 | 0.008 |
|  | Non-LD | 0.81 ± 0.03 | 0.78 ± 0.04 |  |  |  |  |  |  |  |  |  |
| R crus II of the cerebellar hemisphere | LD | 0.90 ± 0.08 | 0.88 ± 0.08 | 2.129 | 0.149 | 0.030 | 0.219 | 0.641 | 0.003 | 1.311 | 0.256 | 0.019 |
|  | Non-LD | 0.85 ± 0.10 | 0.88 ± 0.07 |  |  |  |  |  |  |  |  |  |
| L middle occipital gyrus | LD | 1.16 ± 0.08 | 1.14 ± 0.10 | 7.310 | 0.009 | 0.097 | 0.004 | 0.947 | <0.001 | 0.210 | 0.648 | 0.003 |
|  | Non-LD | 1.21 ± 0.09 | 1.22 ± 0.12 |  |  |  |  |  |  |  |  |  |
| L superior temporal gyrus | LD | 0.93 ± 0.05 | 0.91 ± 0.07 | 4.108 | 0.047 | 0.057 | 0.370 | 0.545 | 0.005 | 0.582 | 0.448 | 0.008 |
|  | Non-LD | 0.95 ± 0.07 | 0.96 ± 0.07 |  |  |  |  |  |  |  |  |  |
| R middle temporal gyrus | LD | 1.00 ± 0.05 | 1.00 ± 0.06 | 7.923 | 0.006 | 0.104 | 0.355 | 0.553 | 0.005 | 1.127 | 0.292 | 0.016 |
|  | Non-LD | 1.03 ± 0.07 | 1.05 ± 0.07 |  |  |  |  |  |  |  |  |  |
| **VBM** |  |  |  |  |  |  |  |  |  |  |  |  |
| R supplementary motor area | LD | 0.33 ± 0.04 | 0.32 ± 0.03 | 8.099 | 0.006 | 0.106 | 1.464 | 0.231 | 0.021 | <0.001 | 0.992 | <0.001 |
|  | Non-LD | 0.36 ± 0.03 | 0.35 ± 0.04 |  |  |  |  |  |  |  |  |  |
| R median cingulate and paracingulate gyri | LD | 0.44 ± 0.03 | 0.42 ± 0.04 | 7.924 | 0.006 | 0.104 | 0.454 | 0.503 | 0.007 | 1.422 | 0.237 | 0.020 |
|  | Non-LD | 0.45 ± 0.04 | 0.46 ± 0.04 |  |  |  |  |  |  |  |  |  |
| L superior occipital gyrus | LD | 0.31 ± 0.03 | 0.31 ± 0.03 | 7.160 | 0.009 | 0.095 | 0.047 | 0.829 | 0.001 | 0.035 | 0.852 | 0.001 |
|  | Non-LD | 0.33 ± 0.05 | 0.33 ± 0.03 |  |  |  |  |  |  |  |  |  |
| L supramarginal gyrus | LD | 0.36 ± 0.03 | 0.35 ± 0.03 | 15.246 | <0.001 | 0.183 | 0.373 | 0.544 | 0.005 | 4.078 | 0.047 | 0.057 |
|  | Non-LD | 0.38 ± 0.04 | 0.40 ± 0.04 |  |  |  |  |  |  |  |  |  |
| L superior temporal gyrus | LD | 0.41 ± 0.03 | 0.40 ± 0.03 | 15.353 | <0.001 | 0.184 | 0.055 | 0.815 | 0.001 | 1.003 | 0.320 | 0.015 |
|  | Non-LD | 0.44 ± 0.05 | 0.45 ± 0.03 |  |  |  |  |  |  |  |  |  |
| **FC** |  |  |  |  |  |  |  |  |  |  |  |  |
| FC1 | LD | 0.11 ± 0.09 | 0.10 ± 0.08 | 0.043 | 0.835 | 0.001 | 0.045 | 0.832 | 0.001 | 0.047 | 0.829 | 0.001 |
|  | Non-LD | 0.11 ± 0.10 | 0.11 ± 0.08 |  |  |  |  |  |  |  |  |  |
| FC2 | LD | 0.19 ± 0.12 | 0.19 ± 0.09 | 0.623 | 0.433 | 0.009 | 0.441 | 0.509 | 0.006 | 0.573 | 0.452 | 0.008 |
|  | Non-LD | 0.23 ± 0.09 | 0.19 ± 0.06 |  |  |  |  |  |  |  |  |  |
| FC3 | LD | 0.10 ± 0.11 | 0.08 ± 0.07 | 2.950 | 0.090 | 0.042 | 0.258 | 0.613 | 0.004 | 0.020 | 0.887 | <0.001 |
|  | Non-LD | 0.13 ± 0.08 | 0.12 ± 0.06 |  |  |  |  |  |  |  |  |  |
| **Brain volumetrics** |  |  |  |  |  |  |  |  |  |  |  |  |
| GMV | LD | 653.12 ± 37.95 | 635.99 ± 37.51 | 13.947 | <0.001 | 0.170 | 0.222 | 0.639 | 0.003 | 1.628 | 0.206 | 0.023 |
|  | Non-LD | 677.24 ± 54.27 | 685.14 ± 31.70 |  |  |  |  |  |  |  |  |  |
| **Hormone** |  |  |  |  |  |  |  |  |  |  |  |  |
| Cortisol | LD | 11.09 ± 5.64 | 16.45 ± 10.39 | 1.808 | 0.183 | 0.026 | 0.022 | 0.882 | <0.001 | 5.260 | 0.025 | 0.072 |
|  | Non-LD | 13.17 ± 14.33 | 8.47 ± 5.81 |  |  |  |  |  |  |  |  |  |
| **Immune** |  |  |  |  |  |  |  |  |  |  |  |  |
| Cluster 22 [CD4^+^ T_CM_ (CD25^+^)] | LD | 2.77 ± 1.25 | 3.33 ± 1.47 | 2.628 | 0.110 | 0.037 | 5.173 | 0.026 | 0.071 | 0.414 | 0.522 | 0.006 |
|  | Non-LD | 3.11 ± 1.36 | 4.11 ± 1.60 |  |  |  |  |  |  |  |  |  |
| Perforin expression in cluster 11 [CD4^-^ CD8^-^ T cells (CD127^+^)] | LD | 0.31 ± 0.37 | 0.46 ± 0.51 | 6.531 | 0.013 | 0.088 | 1.664 | 0.201 | 0.024 | 0.168 | 0.683 | 0.002 |
|  | Non-LD | 0.12 ± 0.20 | 0.20 ± 0.30 |  |  |  |  |  |  |  |  |  |

The continuous data were expressed as the mean ± SD.

FC1, FC2, and FC3 represented the voxel-wise FC for the seed region in the left middle occipital gyrus with clusters in the right triangular part of the inferior frontal gyrus, the voxel-wise FC for the seed region in the left superior occipital gyrus with clusters in the right inferior occipital gyrus, and the voxel-wise FC for the seed region in the right median cingulate and paracingulate gyri with clusters in the right orbital part of the superior frontal gyrus, respectively.

SD, standard deviation; LD, late HIV diagnosis; MRI, magnetic resonance imaging; ALFF, amplitude of low-frequency fluctuations; ReHo, regional homogeneity; VBM, voxel-based morphometry; FC, functional connectivity; GMV, gray matter volume; L, left; R, right; T_CM_, T central memory cells.**Supplementary Table 11.** Interaction effects of groups and neuropsychiatric disorders on multidimensional biomarkers.

| **Biomarker** | **Analysis Dimension** | **Group Comparison** | **Mean Difference (95% CI)** | ***F*** | ***P*** | **Partial η²** |
| --- | --- | --- | --- | --- | --- | --- |
| **ALFF** |  |  |  |  |  |  |
| L lobule VIII of the cerebellar hemisphere | Main Effect of Groups |  |  |  |  |  |
|  | Negative state | Non-LD vs LD | -0.009 (-0.070, 0.051) | 0.097 | 0.756 | 0.001 |
|  | Positive state | Non-LD vs LD | -0.108* (-0.175, -0.042) | 10.566 | 0.002 | 0.134 |
|  | Main Effect of Neuropsychiatric Disorders |  |  |  |  |  |
|  | Non-LD group | Negative vs Positive | 0.016 (-0.048, 0.079) | 0.239 | 0.626 | 0.004 |
|  | LD group | Negative vs Positive | -0.083* (-0.146, -0.020) | 6.917 | 0.011 | 0.092 |
| **GMV** |  |  |  |  |  |  |
| L supramarginal gyrus | Main Effect of Groups |  |  |  |  |  |
|  | Negative state | Non-LD vs LD | 0.017 (-0.007, 0.040) | 1.967 | 0.165 | 0.028 |
|  | Positive state | Non-LD vs LD | 0.052* (0.026, 0.078) | 15.997 | <0.001 | 0.19 |
|  | Main Effect of Neuropsychiatric Disorders |  |  |  |  |  |
|  | Non-LD group | Negative vs Positive | -0.023 (-0.048, 0.002) | 3.423 | 0.069 | 0.048 |
|  | LD group | Negative vs Positive | 0.012 (-0.012, 0.037) | 1.003 | 0.32 | 0.015 |
| **Hormone** |  |  |  |  |  |  |
| Cortisol | Main Effect of Groups |  |  |  |  |  |
|  | Negative state | Non-LD vs LD | 2.081 (-3.802, 7.964) | 0.498 | 0.483 | 0.007 |
|  | Positive state | Non-LD vs LD | -7.981* (-14.464, -1.497) | 6.034 | 0.017 | 0.081 |
|  | Main Effect of Neuropsychiatric Disorders |  |  |  |  |  |
|  | Non-LD group | Negative vs Positive | 4.703 (-1.519, 10.924) | 2.275 | 0.136 | 0.032 |
|  | LD group | Negative vs Positive | -5.359 (-11.519, 0.800) | 3.015 | 0.087 | 0.042 |

*indicates significance after Bonferroni correction (*P* < 0.05). Mean difference values reflect pairwise comparisons (I–J), where I represents the reference group and J denotes the comparison group. Positive values indicate greater measurements in the reference group (I).

ALFF: Amplitude of Low-Frequency Fluctuations; GMV, gray matter volume; LD, late HIV diagnosis; L, left.

**Supplementary Table 12.** Gray matter volume of the left supramarginal gyrus for each participant in the LD and LD groups.

| LD group | | | |  | Non-LD group | |  |  |
| --- | --- | --- | --- | --- | --- | --- | --- | --- |
| Numbering | GMV | Numbering | GMV |  | Numbering | GMV | Numbering | GMV |
| sub001 | 0.3533 | sub021 | 0.3977 |  | sub001 | 0.379 | sub021 | 0.4625 |
| sub002 | 0.3461 | sub022 | 0.3492 |  | sub002 | 0.4356 | sub022 | 0.3616 |
| sub003 | 0.3414 | sub023 | 0.3526 |  | sub003 | 0.3851 | sub023 | 0.4023 |
| sub004 | 0.378 | sub024 | 0.3786 |  | sub004 | 0.4696 | sub024 | 0.3561 |
| sub005 | 0.3833 | sub025 | 0.4299 |  | sub005 | 0.3518 | sub025 | 0.3293 |
| sub006 | 0.3085 | sub026 | 0.335 |  | sub006 | 0.4251 | sub026 | 0.4303 |
| sub007 | 0.3789 | sub027 | 0.3682 |  | sub007 | 0.4008 | sub027 | 0.3623 |
| sub008 | 0.3403 | sub028 | 0.341 |  | sub008 | 0.3888 | sub028 | 0.4432 |
| sub009 | 0.371 | sub029 | 0.3722 |  | sub009 | 0.3924 | sub029 | 0.4162 |
| sub010 | 0.3478 | sub030 | 0.4141 |  | sub010 | 0.4303 | sub030 | 0.3389 |
| sub011 | 0.4081 | sub031 | 0.3602 |  | sub011 | 0.3885 | sub031 | 0.3492 |
| sub012 | 0.3738 | sub032 | 0.31 |  | sub012 | 0.3572 | sub032 | 0.3709 |
| sub013 | 0.3209 | sub033 | 0.3465 |  | sub013 | 0.3248 | sub033 | 0.4929 |
| sub014 | 0.3282 | sub034 | 0.3771 |  | sub014 | 0.3746 | sub034 | 0.3588 |
| sub015 | 0.3199 | sub035 | 0.348 |  | sub015 | 0.3598 |  |  |
| sub016 | 0.3996 | sub036 | 0.4012 |  | sub016 | 0.4235 |  |  |
| sub017 | 0.3187 | sub037 | 0.2917 |  | sub017 | 0.3879 |  |  |
| sub018 | 0.3657 | sub038 | 0.3721 |  | sub018 | 0.3656 |  |  |
| sub019 | 0.3926 |  |  |  | sub019 | 0.4449 |  |  |
| sub020 | 0.3334 |  |  |  | sub020 | 0.4037 |  |  |

Abbreviations: LD, late HIV diagnosis; GMV, gray matter volume.


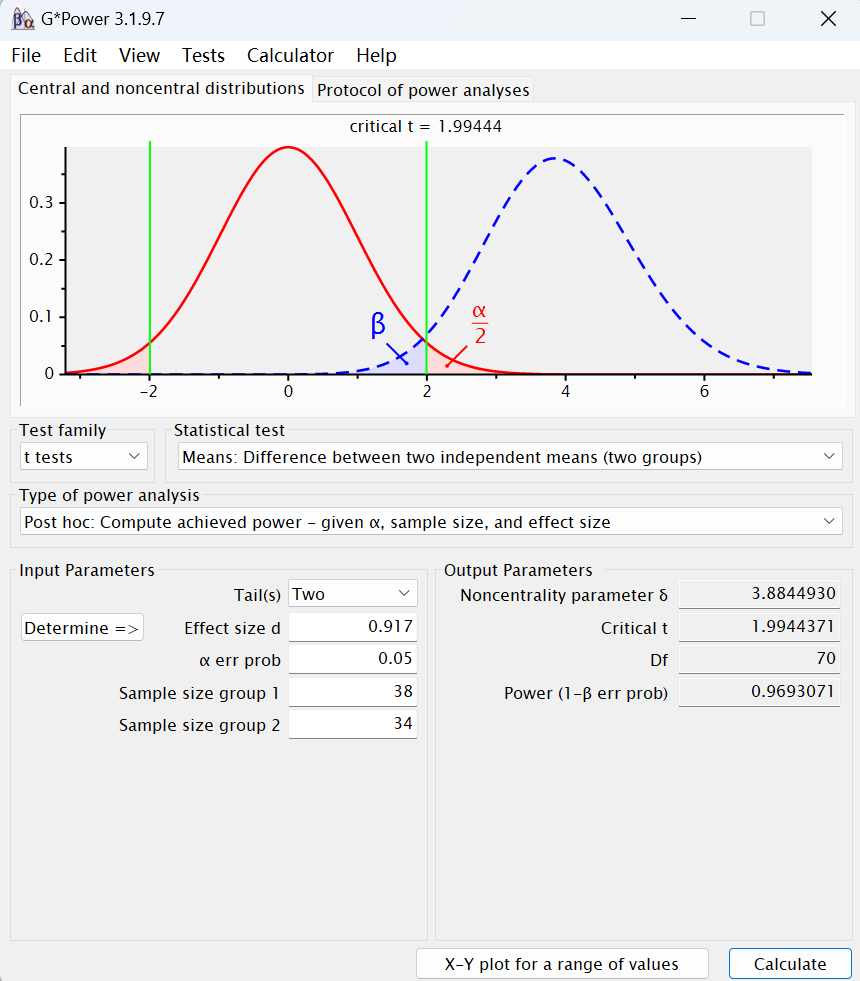


**Supplementary Figure 4.** Post-hoc power analysis calculated using the statistical software G*Power.
